# Analysis of the key ligand receptor CADM1_CADM1 in the regulation of thyroid cancer based on scRNA-seq and bulk RNA-seq data

**DOI:** 10.3389/fendo.2022.969914

**Published:** 2022-11-29

**Authors:** Hui He, Shan Cong, Yu Wang, Qinghai Ji, Weiyan Liu, Ning Qu

**Affiliations:** ^1^ Department of Head and Neck Surgery, Fudan University Shanghai Cancer Center, Shanghai, China; ^2^ Department of Oncology, Shanghai Medical College, Fudan University, Shanghai, China; ^3^ Department of Laparoscopic Surgery, the First Affiliated Hospital of Dalian Medical University, Dalian, Liaoning, China; ^4^ Department of General Surgery, Institute of Fudan-Minhang Academic Health System, Minhang Hospital, Fudan University, Shanghai, China

**Keywords:** advanced PTC, ligand-receptor pair, CADM1_CADM1, targeted therapy, prognostic biomarker

## Abstract

**Introduction:**

Advanced papillary thyroid cancer (PTC) has a poor prognosis, 60~70% of which become radio iodine refractory (RAI-R), but the molecular markers that assess PTC progress to advanced PTC remain unclear. Meanwhile, current targeted therapies are badly effective due to drug resistance and adverse side effects. Ligand-receptor pairs (L/R pairs) play an important role in the interactions between tumor cells and other cells in the tumor microenvironment (TME). Nowadays, therapies targeting ligand-receptor pairs in the TME are advancing rapidly in the treatment of advanced cancers. However, therapies targeting L/R pairs applied to advanced PTC remains challenging because of limited knowledge about L/R pairs in PTC.

**Methods:**

We screened the critical L/R pair: CADM1-CADM1 using 65311 single-cell RNA sequencing (scRNA-seq) samples from 7 patients in different stage of PTC and bulk RNA-seq datasets containing data from 487 tumor samples and 58 para-carcinoma samples. Moreover, the expression levels of CADM1-CADM1 was assessed by quantitative real time polymerase chain reaction (qRT-PCR) and the function was analyzed using Transwell immigration assay.

**Results:**

We found that CADM1_CADM1 could be regarded as a biomarker representing a good prognosis of PTC. In addition, the high expression of CADM1_CADM1 can strongly increase the sensitivity of many targeted drugs, which can alleviate drug resistance. And the results of qRT-PCR showed us that the expression of CADM1_CADM1 in PTC was down-regulated and overexpression of CADM1 could suppresses tumor cell invasion migration.

**Conclusion:**

Our study identified that CADM1_CADM1 played an essential role in the progression of PTC for the first time and our findings provide a new potential prognostic and therapeutic ligand-receptor pair for advanced PTC.

## 1 Introduction

Thyroid cancer (TC) is the most common endocrine system tumor, which has increased year by year in recent years. In 2020, the number of new thyroid cancer cases in the world has reached about 580,000 (1, 2). PTC is the most common TC, which has a good prognosis with a ten-year survival rate of 90% through surgery, radioactive iodine (RAI) ablation and thyroid stimulating hormone (TSH) suppression (3). However, local recurrences occur in about 20% of PTC and distant metastases in 10%, more commonly in the lung (50%) and bone (25%) (4). These PTC are called advanced thyroid cancer, even 60%~70% of which become RAI-R with a mean life expectancy of 3–5 years (5). At present, systemic and targeted therapies have been used to treat these PTC patients, such as lenvantinib, sorafenib and cabozantinib. However, its therapeutic effect is not ideal due to the emergence of drug resistance and adverse side effects (6, 7). Thus, it is urgently required to find more tumor markers to reflect the different physiological development processes within the tumor and explore novel therapeutic targets and approaches for advanced thyroid cancer.

In recent years, more and more studies show that TME plays an important role in the occurrence and development of tumors. The TME refers to the cellular environment in which tumors exist, which encompasses the surrounding immune cells, blood vessels, extracellular matrix (ECM), fibroblasts, lymphocytes, bone marrow‐derived inflammatory cells, and signaling molecules (8–10). This cellular complexity of tumors is further increased by the heterogeneity of each cell type, such as different clones of tumor cells or the various subsets of immune cells (11). Cell-to-cell communication across multiple cell types and tissues extensively relies on interactions between secreted ligands and cell-surface receptors or exosomes (12). Cell-to-cell communication based on ligand-receptor interactions play an essential role in the mechanisms of oncogenesis, tumor progression, therapeutic resistance, immune infiltration, and inflammation (13). At present, targeted therapy based on the ligand-receptor interaction (LRI) has been well-developed and used in some tumors, such as colorectal cancer (14). However, there is a scarcity of studies about LRI associated with PTC.

With the development of sequencing, single-cell RNA sequencing (SC-RNA-seq) appeared, which are cell specific towards investigating cellular functionalities of DNA and RNA in different cellular subsets (15). At present, SC-RNA-seq has been widely used in different types of tissues and cell lines of various species (especially human and mouse), including normal and pathological cells. SC-RNA-seq can reveal the expression of all genes in the whole genome at the single cell level, which makes it possible to deeper explore LRIs.

In this study, we screened the critical L/R pair: CADM1_CADM1 by analyzing scRNA-seq data from human PTC samples and bulk RNA-seq data in TCGA. Next, we analyzed the relationship between CADM1-CADM1 and clinical characteristics, further discussed pathways regulated by CADM1-CADM1 and biological processes associated with CADM1-CADM1. In addition, we identified its value for targeted therapies.

## 2 Materials and methods

### 2.1 Collection of data

#### 2.1.1 Collection of data from single cell

The GSE184362 complete dataset (https://www.ncbi.nlm.nih.gov/geo/query/acc.cgi?acc=GSE184362) was available in Gene Expression Omnibus (GEO). We only chose scRNA-seq data from 7 PTC patients: PTC1, PTC2, PTC3, PTC5, PTC8, PTC9, PTC10.

#### 2.1.2 Collection of data from bulk tissue

The bulk RNA-seq profiles were integrated from the TCGA dataset (https://portal.gdc.cancer.gov). The FPKM data of thyroid cancer tumor samples was further processed into TPM data. After eliminating samples without survival time and survival state, we finally got clinical information of 487 tumor samples and 58 para-cancer tissue samples.

#### 2.1.3 Collection of gene data associated with cell death

27 genes related to pyroptosis were downloaded from the msigdb database of GSEA (http://www.zhounan.org/ferrdb). 111 ferroptosis-related genes were obtained from the FerrDb database. 74 apoptosis-related genes were downloaded from a previous study (https://www.frontiersin.org/articles/10.3389/fgene.2022.832046/full). 279 cell senescence-related genes were downloaded from the cellAge database (https://genomics.senescence.info/cells/).

#### 2.1.4 Collection of data related to pan-cancer

The gene expression data of pan-cancer was downloaded from sangerbox (http://vip.sangerbox.com). The gene expression data of involved cancers in normal tissue was downloaded in GTEx dataset (https://maayanlab.cloud/Harmonizome/dataset/GTEx+Tissue+Gene+Expression+Profiles).

### 2.2 DNA methylation dataset processing

The dataset of PTC methylation was downloaded in TCGA. In the pretreatment processing we removed CpG sites at which each sample had missing values (NA), cross-reactive CpG sites according to Discovery of cross-reactive probes and polymorphic CpGs in the Illumina Infinium HumanMethylation450 microarray, unstable genomic methylation sites, CpG sites on sex chromosomes and single nucleotide sites but preserved tumor tissue.

### 2.3. Screening of L/R pairs

#### 2.3.1 scRNA−seq data processing and construction of cell clusters

The ‘Seurat’ package was used for quality control procedures by analyzing the matrix of unique molecular identifier (UMI) counts per gene. We selected cells meeting the following criteria: (1) Each gene is expressed in at least 3 cells, and each cell expresses at least 250 genes. (2) Cell expresses with 1000 UMIs at least and more than 100 genes, less than 4000 genes. (3) Less than 25% mitochondrial gene expression in UMI counts. We normalized the data of 7 samples separately using the normalization method “Log Normalize”. Variable genes were selected by FindVariableFeautres. After removing batch effect using Seurat’s Canonical Correlation Analysis (CCA), data was further Integrated using the IntegrateData function. Then, the optimal number of PCs was selected using the PCElbowPlo function in Seurat. We performed the PCElbowPlo function in Seurat to select the optimal number of PCs. We used the FindNeighbors function and FindClusters function in Seurat to perform cell clustering (Resolution=0.1). Next, the RunTSNE function was performed to visualize cell clusters defined by the expression of known marker genes. Finally, we got 8 cell clusters.

#### 2.3.2 Screening of markers for cell clusters

We used the FindAllMarkers function in Seurat to screen the markers for cell clusters, which met the following criteria: (1) |log2FC | > 0.5; (2) Wilcoxon rank-sum test adjusted P value < 0.01; (3) min.pct=0.35. We showed the results by bubble chart. Furthermore, we compared the percent of 7 samples in 8 cell clusters. KEGG pathway enrichment analysis was performed in cell markers of 8 cell clusters using clusterProfiler package.

#### 2.3.3 Identification of crucial L/R pairs

We distinguished malignant cells and normal cells by comparing the copy number variations of cells using the copyKAT package. Next, cell-cell interaction analysis based on L/R pairs was performed by cellchart package. 38 L/R pairs of malignant/non-malignant and 44 L/R pairs of non-malignant/malignant (p<0.05) were found. Then the 38 L/R pairs and 44 L/R pairs were overlapped and 14 L/R pairs were screened. Given that the presence of one ligand corresponding with multiple receptors, we repaired 14 L/R pairs into 16. The t-test was used to compare 16 L/R pairs between tumor tissues and para-tumor tissues in TCGA (p<0.05). Univariate COX regression analysis was performed to analyze the relationship between L/R pairs and prognosis (p<0.05).

### 2.4 GO and KEGG enrichment analysis

To analyze the biological significance of genes associated with crucial L/R pairs, GO term enrichment analysis was applied using clusterProfiler. The function “enrich GO” and “enrich KEGG” with default parameters, were used for the GO terms and KEGG pathway enrichment analyses. FDR < 0.05 was considered statistically significant for both GO and KEGG analysis.

### 2.5 Cell culture and transfection

The human PTC cell lines K1, BCPAP and normal TC cell N3 were provided by Science Experimental Center of China Medical University. IHH4 and TPC1 cell lines were respectively acquired from Shanghai Whelab Bioscience Limited and Procell Life Science & Technology Co, Ltd. The cells were cultured in 1640 medium (Invitrogen, Carlsbad, CA) containing 10% fetal calf serum and incubated in a humidified atmosphere of 5% CO2 at 37°C. The eukaryotic expression vector pEGFP-C1-CADM1 was purchased from Genepharma (Shanghai, China). The human thyroid cancer cell lines were plated at a density of 4 × 10^4^ cells per well in 24-well culture plates. The IHH4 and TPC1 cells were cultured overnight and then transfected with the expression vector pEGFP-C1-CADM1 or pEGFP-C1 using the Lipofectamine 3000 reagent (Invitrogen) according to the manufacturer’s recommendation. CADM1 expression was confirmed by a Western blot analysis.

### 2.6 RNA isolation and real-time polymerase chain reaction

The total RNA was isolated from the frozen tumor specimens with a TRIzol reagent (Takara, Otsu, Japan) according to the manufacturer’s instructions. Reverse transcription was performed using 0.5 mg total RNA from each sample. qRT-PCR was performed using the SYBR Green PCR Master Mix (Takara). The sequences of the primer pairs were as follows: CADM1 (forward), 5’- GTCCCACCACGTAATCTGATG-3’; CADM1 (reverse), 5’- CCACCTCCGATTTGCCTTTTA-3’; GAPDH (forward), 5’-GGAGCGAGATCCCTCCAAAAT-3’; and GAPDH (reverse), 5’-GGCTGTTGTCATACTTCTCATGG-3’. The experiments were repeated in triplicate. The relative levels of gene expression were represented as ΔCt = Ct^CADM1^ – Ct^GAPDH^, and the fold change of gene expression was computed using the 2^–ΔΔCt^ method.

### 2.7 Immunofluorescence

Cells were spread in a six-well plate with an appropriate number of monolayer cells, and washed twice with PBS the next day. Cells were fixed with 4% paraformaldehyde for 15min and washed with PBS three times, 3mim each time. Permeable: 1% Triton X-100 permeable with 10mim PBS was washed 3 times with 3min each time. Block: 5%BSA block 30mim. Primary antibodies (CADM1, Immunoway, YT4764, 1:300) were incubated and placed in a wet box at 4°C overnight. The primary antibody was recovered and washed 3 times with 5min each time with PBS. Incubation with fluorescent secondary antibody: diluted secondary antibody was incubated in a wet box for 1h, and washed 3 times with 5min each time with PBS. Avoid light for all subsequent steps. Double check staining: add DAPI drop, incubate for 5min in the dark, and wash off excess dye. Fluorescence microscope photography.

### 2.8 Transwell immigration assay

Preparation of cell suspension: Starved cells were treated for 12h and digested, then resuspended with serum-free medium. Inoculated cells: 200ul of cell suspension, cells (5 × 10^4^) were inoculated into the upper chamber, and 600ul of medium containing 15% fetal bovine serum was added into the lower chamber, and the cells were checked 24 hours later. Fixation staining: Remove the chamber, discard the culture medium, wash twice with PBS, gently wipe the upper layer of the cotton swab for migrating cells, fix with 4% paraformaldehyde for 30min, wash twice with PBS, and wipe off the moisture on the cotton swab. Count.

### 2.9 Statistical analysis

Cox regression models were used to assess the relationship between gene module scores and prognosis (OS, DFS and PFI), and p <0.05 was the significance threshold in the log-rank test. Wilcox rank test was used to test the significance between two groups of continuous variables, while Kruskal Wallis rank test was used for the significance test of more than two groups. The BH method calculated the false discovery rate (FDR). All of the above analysis was done using R (version 3.6.3). Unless otherwise specified, * * * represented p <1×e-5, * * represented p <0.01 and * represented p <0.05.

## 3 Results

### 3.1 Construction of cell clusters in PTC using scRNA−seq data

**Figure 1:**
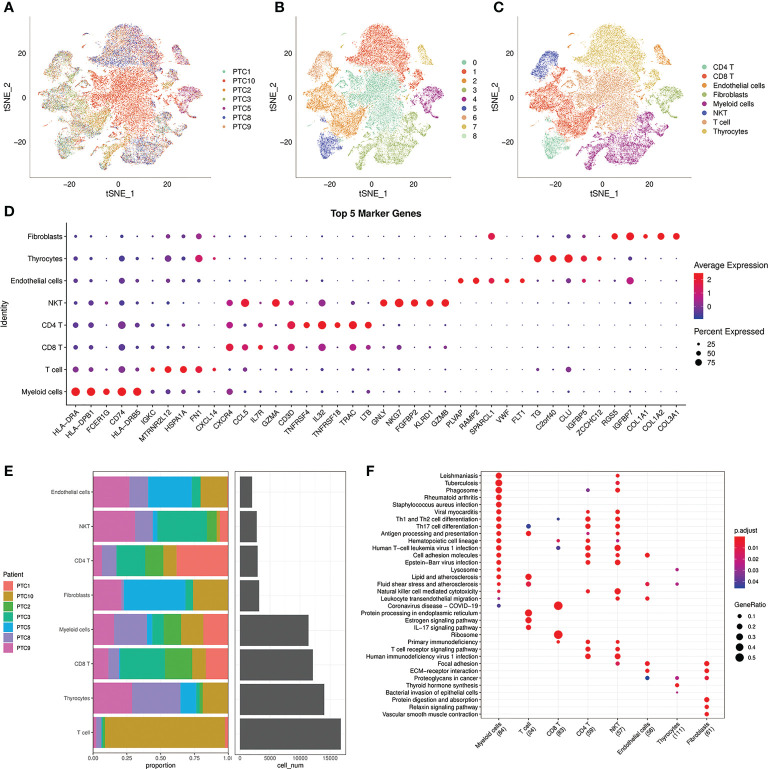
Analysis of 8 cell lineages. **(A)** t-SNE plot of filtered cells colored by samples. **(B)** t-SNE plot of filtered cells colored by clusters. **(C)** The cell lineages were identified by marker genes: CD4 T, CD8 T, endothelial cells, fibroblasts, myeloid cells, NK T, T cells, thyrocytes. **(D)** The expression of the first 5 marker genes in each cell lineages. The size of the dot represents the fraction of cells in which the gene was detected. The color of the dot represents the average expression of the cells in each lineage. **(E)** The proportion of cells from 7 PTC samples in 8 cell lineages. **(F)** KEGG enrichment analysis of 8 cell lineages.

For the reason that genes, cells and signaling molecules dynamically alter throughout tumor evolution, we used scRNA-seq to profile cells of 7 patients in different stage of PTC: PTC9 is intrathyroidal tumor; PTC2,3 are intrathyroidal tumor with lateral neck metastasis; PTC1 is tumor with extrathyroidal extension; PTC8 is tumor with extrathyroidal extension and lateral neck metastasis; PTC5,10 are tumor with distant metastasis and PTC5 is follicular vessel papillary thyroid cancer (FVPTC). There are 65311 cells incorporated in further analysis after the stringent quality filtering (**Supplementary Figure 1**; **Table 1**). Based on the transcriptional data from all acquired cells, we distributed all cells into 9 cell clusters using t-distributed stochastic neighbor embedding (t-SNE) (**Figures 1A, B**). Furthermore, we applied known cell markers to annotate 9 cell clusters (**Supplementary Figure 2**) and got 8 cell types: CD4 T, CD8 T, endothelial cells, fibroblasts, myeloid cells, NK T, T cells, thyrocytes in the final (**Figure 1C**). After analyzing the distribution of 7 PTC samples in 8 cell types (**Figure 1E**), we can see that the percentage of each cell type changed with the progress of the tumor. Under this circumstance, we screened cell markers of 8 cell types (**Figure 1D**) and performed KEGG Enrichment Analysis (**Figure 1F**). NK T cells with natural killer cell mediated cytotoxicity mainly occurred in the early stage of the tumor PTC9, which control tumor growth and mediate a robust anti-metastatic effect (16). On the contrary, T cells would increase protein processing in endoplasmic reticulum and estrogen signaling pathway during advanced stage of tumor PTC10, which promotes primary tumor growth and distant metastasis and increase the expression of MET proto-oncogene (17, 18). In the meanwhile, PTC5, as FVPTC in advanced stage of tumor, fibroblasts played an important role in enhancing tumor cell adhesion and promoting tumor growth and metastasis ability (19), which occupied most of the PTC5 cells. Together, TME and tumor interact and influence each other, jointly determining the progression of the tumor.

**Table 1:** Number of cells before and after filtration of the samples.

Sample	raw_count	clean_count	percent %
PTC1	5917	5896	99.65
PTC10	22351	22196	99.31
PTC2	5100	5097	99.94
PTC3	7433	7430	99.96
PTC5	4475	4463	99.73
PTC8	10519	10354	98.43
PTC9	10065	9875	98.11

### 3.2 Screening of crucial L/R pairs

**Figure 2:**
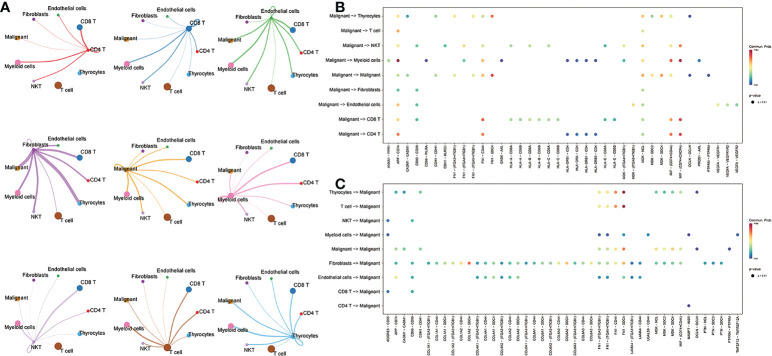
The L/R pairs between malignant cells and normal cells. **(A)** The ligand-receptor interactions among cells. Thicker lines represent more interactions (more ligand receptors). **(B)** Bubble plot of L/R pairs communicating between ligands of malignant cells and receptors of non-malignant cells; **(C)** Bubble plot of L/R pairs communicating between ligands of non-malignant cells and receptors of malignant cells; the color represents the magnitude of the force, and the larger the point, the smaller the p-value (p<0.05).

**Figure 3:**
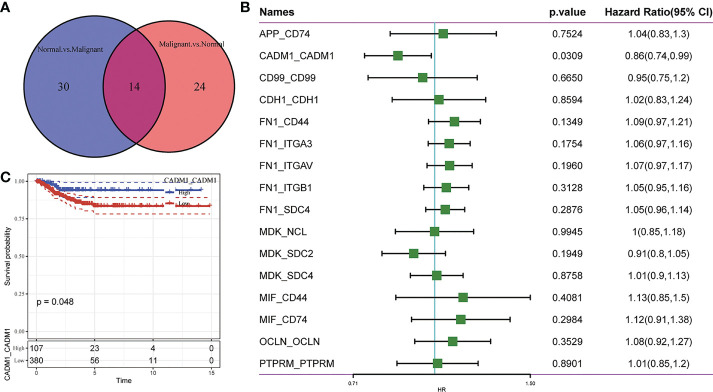
Screening of crucial L/R pairs. **(A)** Venn diagram of 38 L/R pairs communicating between malignant cells/non-malignant cells and 44 L/R pairs communicating between non-malignant cells/malignant cells. **(B)** Forest plot of univariate Cox regression analysis of 16 L/R pairs. **(C)** KM curves of CADM1-CADM1.

To further understand the interactions between tumor cells and TME, we integrated and screened crucial L/R pairs (p< 0.05) between tumor cells and normal cells in the TME through cellchart package in R. We found 38 L/R pairs in malignant/non-malignant cells and 44 L/R pairs in non-malignant/malignant cells (**Figure 2**). Next, they were overlapped (**Figure 3A**) and re-paired due to the presence of one ligand corresponding with multiple receptors. 16 L/R pairs: *MIF-CD74, MIF-CD44, MDK-SDC2, MDK-SDC4, MDK-NCL, FN1-ITGA3, FN1-ITGB1, FN1-ITGAV, FN1-CD44, FN1-SDC4, APP-CD74, CADM1-CADM1, CD99-CD99, CDH1-CDH1, OCLN-OCLN and PTPRM-PTPRM* (**Supplementary Figure 3**) were finally screened. More specifically, to find the L/R pairs significantly associated with the development of tumor, we further analyzed the crucial L/R pairs in the TCGA dataset by comparing the different expressions of L/R pairs between tumor tissues and para-carcinoma tissues (**Table 2**; **Supplementary Table 1**) and performing univariate cox regression analysis (**Figure 3B**). At last, CADM1-CADM1 was recognized as the only crucial L/R pair (**Supplementary Table 2**), higher expression of which would lead to a better prognosis (HR<1). KM survival analysis showed that overall survival (OS) of high CADM1-CADM1 was longer than low CADM1-CADM1 (**Figure 3C**). Interestingly, CADM1-CADM1 was also essential to PTC of other stage: KM survival analysis showed that disease free interval (DFI) and progression-free interval (PFI)of high CADM1-CADM1 were both longer than low CADM1-CADM1 (**Supplementary Figures 4**, **5**). A present study has shown that for less aggressive cancer types, OS is clearly not a suitable study endpoint because of requiring 10 years or more to recur or death, and disease-free survival (DFS) and PFI are recommended to use without reservation (20). Thus, CADM1-CADM1 was not only useful to analyze the prognosis of advanced PTC but also assess the progression of PTC.

**Table 2:** Differential analysis of 16 L/R pairs.

LR pair	logFC	p.value
CADM1_CADM1	0.0603	0.0473
MDK_SDC2	0.0424	0.0529
PTPRM_PTPRM	0.0495	0.0531
FN1_ITGAV	0.081	0.063
FN1_SDC4	0.0833	0.0727
FN1_ITGB1	0.0738	0.0891
MDK_SDC4	0.0549	0.0955
APP_CD74	0.0018	0.894
CD99_CD99	-0.0259	0.1224
CDH1_CDH1	0.0179	0.302
FN1_CD44	0.0502	0.2136
FN1_ITGA3	0.073	0.1128
MDK_NCL	0.0232	0.2927
MIF_CD44	-0.0139	0.3831
MIF_CD74	-0.0122	0.4823
OCLN_OCLN	0.0654	0.1705

### 3.3 The expression of CADM1-CADM1 in the progression of PTC

**Figure 4:**
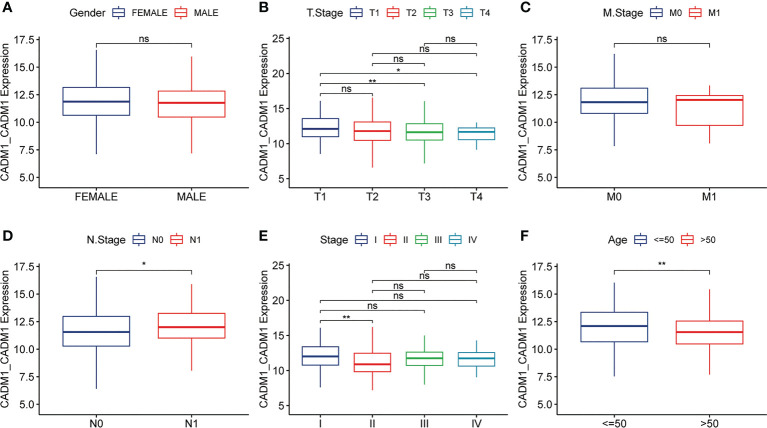
Differences in the expression of CADM1-CADM1 in clinical phenotypes (** P < 0.01, *P < 0.05). The differences in CADM1-CADM1 by gender **(A)**, T stage **(B)**, M stage **(C)**, N stage **(D)**, stage **(E)** and age **(F)**. ns, no significance.

As mentioned earlier, CADM1-CADM1 is related to good prognostic outcomes. To better explore the influence of CADM1-CADM1 on the progression of PTC, we compared the expression of CADM1-CADM1 in different clinical features (**Figure 4**). We found CADM1-CADM1 was strongly correlated with tumor size, extrathyroidal extension, lymph node metastasis (LNM) and age. CADM1-CADM1 was more highly expressed in T1 than T3 and T4, when primary tumor is ≤2cm and intrathyroidal tumor. CADM1-CADM1 was also more highly expressed in age<50 and stage I than stage II and age>50. These results all proved that CADM1-CADM1 was associated with better clinical features. However, CADM1-CADM1 was more highly expressed in N1 than N0. LNM is a complex process that involves multiple gene variations, or dysregulation and activation of multiple signaling pathways (21). The expression of CADM1-CADM1 may regulated by many genes and signaling pathways. Although we can’t illustrate CADM1-CADM1 completely inhibit the development of tumor, the high expression of it may reduce tumor proliferation and invasion.

### 3.4 CADM1-CADM1 function enrichment analysis

**Figure 5:**
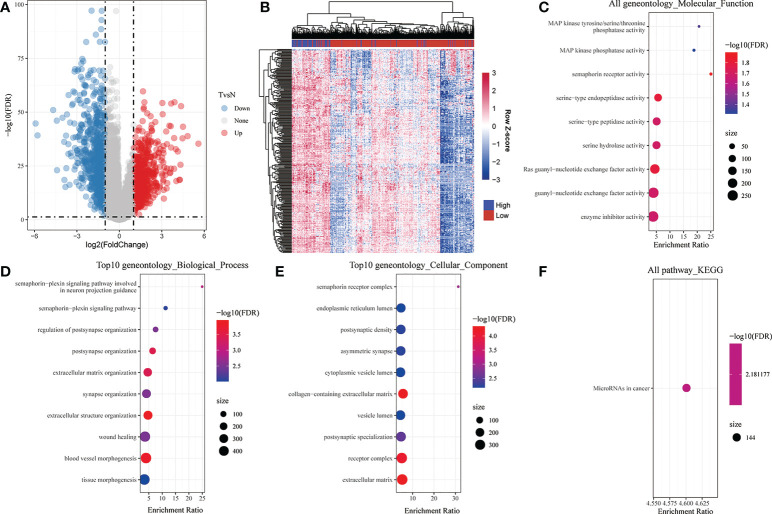
Analysis of differential genes associated with CADM1-CADM1. **(A)** Volcano map showing differential genes between tumor tissues and para-tumors. **(B)** Bidirectional Clustering Heatmap showing the relationship between CADM1-CADM1 and differential genes associated with CADM1-CADM1. Each column in the heatmap represents a sample, and each row represents the expression level of a gene. The color scale beside the heatmap represents the raw Z-score: blue (low expression), red (high expression). **(C)** Scatter plot of 9 enriched GO terms of molecular function (MF). **(D)** Scatter plot of top 10 enriched GO terms of biological process (BP). **(E)** Scatter plot of top 10 enriched GO terms of cellular component (CC). **(F)** KEGG enrichment analysis of differential genes associated with CADM1-CADM1.

To comprehensively understand the function of CADM1-CADM1, we screened 281 differential genes associated with CADM1-CADM1 from 1421 genes differentially expressed in cancer tissues and normal tissues (**Figure 5A**; **Supplementary Table 3**), which was screened by the threshold: the |log2 fold change (FC)| >1 and p-value < 0.05. They included 279 positively correlated genes and 2 negatively correlated genes (**Figure 5B**; **Supplementary Table 4**). GO and KEGG pathway enrichment analyses were performed to explore the functional characteristics of 281 genes. The GO analysis results revealed that these genes were significantly enriched in 9 functions such as ‘Ras guanyl-nucleotide exchange factor activity’, ‘serine-type endopeptidase activity’, ‘enzyme inhibitor activity’ in terms of MF (**Figure 5C**). Regarding BP, these genes were enriched in 55 functions such as ‘blood vessel morphogenesis’, ‘extracellular matrix organization’, ‘extracellular structure organization’ (**Figure 5D**). Under CC, these genes were enriched in 41 functions such as ‘collagen-containing extracellular matrix’, ‘receptor complex’, ‘extracellular matrix’ (**Figure 5E**). In addition, the KEGG analysis results showed that these genes were only significantly enriched in ‘MicroRNA in cancer’ pathway (**Figure 5F**), which play an important role in tumor onset, growth, and metastasis due to its extensive deregulation (22). The detailed information of KEGG and GO enrichment results are listed in **Supplementary Text** (**Supplementary Table 5**).

### 3.5 Analyzing the molecular mechanisms of tumor progression regulated by CADM1-CADM1

**Figure 6:**
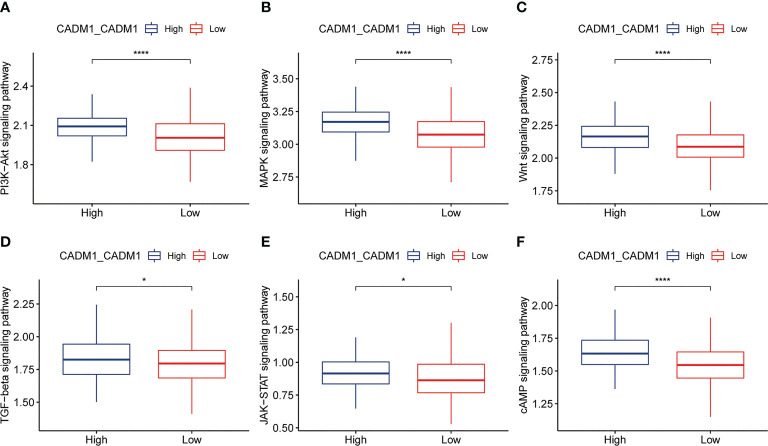
Box plots showing ssGSEA scores of related signaling pathways in high and low expression groups of CADM1-CADM1 (****P < 0.0001, *P < 0.05). **(A)** PI3K-Akt signaling pathway. **(B)** MAPK signaling pathway. **(C)** Wnt signaling pathway. **(D)** TGF-β signaling pathway. **(E)** JAK-STAT signaling pathway. **(F)** cAMP signaling pathway.

Based on the previous analysis, we have known that CADM1-CADM1 mainly distributed in malignant-malignant, malignant-thyrocytes, thyrocytes-malignant, which meant CADM1 can trigger intracellular signal transduction to play a role. S. Murakami et al. (23) demonstrated the same distribution of CADM1-CADM1 (**Table 3**; **Supplementary Table 6**). Previous studies have shown this intracellular signaling generally determines the triggering of MAPKs and of PI3K/Akt/mTOR pathways to take part in the progression of tumor (24). Through comparing the differences of ssGSEA score of signaling pathways in high/low CADM1-CADM1, including PI3K-Akt signaling pathway, MAPK signaling pathway, Wnt signaling pathway, TGF-β signaling pathway, JAK-STAT signaling pathway and cAMP signaling pathway, we found high expression of CADM1-CADM1 would significantly modulate these pathways especially PI3K-Akt signaling pathway, MAPK signaling pathway, Wnt signaling pathway and cAMP signaling pathway (**Figure 6**). Based on previous studies, CADM1-CADM1 can suppress the progression of tumor through upregulating or downregulating associated pathways.

**Table 3:** Information of pathways involved in CADM1-CADM1.

source	target	LR	pathway	annotation	evidence
Malignant	Malignant	CADM1_CADM1	CADM	Cell-Cell Contact	PMID: 24503895
Malignant	Thyrocytes	CADM1_CADM1	CADM	Cell-Cell Contact	PMID: 24503895
Thyrocytes	Malignant	CADM1_CADM1	CADM	Cell-Cell Contact	PMID: 24503895

### 3.6 The connection between CADM1-CADM1 and DNA, RNA methylation

**Figure 7:**
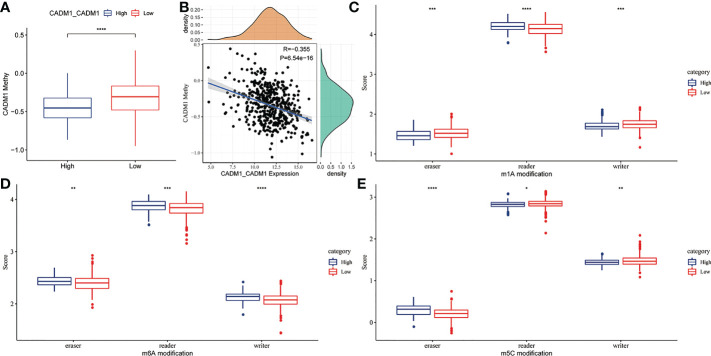
The relationship between CADM1-CADM1 and DNA, RNA methylation. **(A)** Comparison of CADM1 methylation in high CADM1-CADM1 group and low CADM1-CADM1 group. **(B)** Correlation analysis of CADM1-CADM1 and CADM1 methylation. **(C–E)** Comparison of the scores of erasers, readers, writers in m1a, m6a, m5c modification between high CADM1-CADM1 group and low CADM1-CADM1 group separately (****P < 0.0001, ***P < 0.001, **P < 0.01, *P < 0.05).

DNA and mRNA methylation can cause tumor and promote tumor progression through the regulation of gene expression. To explore whether DNA and RNA methylation regulate the expression of CADM1-CADM1, we compared the degree of DNA and RNA methylation between high CADM1-CADM1 group and low CADM1-CADM1 group. The result showed us the degree of DNA methylation was significantly lower in high CADM1-CADM1 group (**Figure 7A**). What’s more, the expression of CADM1-CADM1 showed a significant negative correlation with the degree of DNA methylation (**Figure 7B**). As for RNA methylation, which was adjusted by three parts: eraser, reader, writer, we analyzed the differences in the three parts of m1a, m6a and m5c modification functions by comparing gene scores based on ssGSEA. We found the expression of CADM1-CADM1 mainly associated with readers in m1a, m6a and m5c and was strongly influenced by RNA methylation (**Figures 7C–E**).

### 3.7 The connection between CADM1-CADM1 and cell death

**Figure 8:**
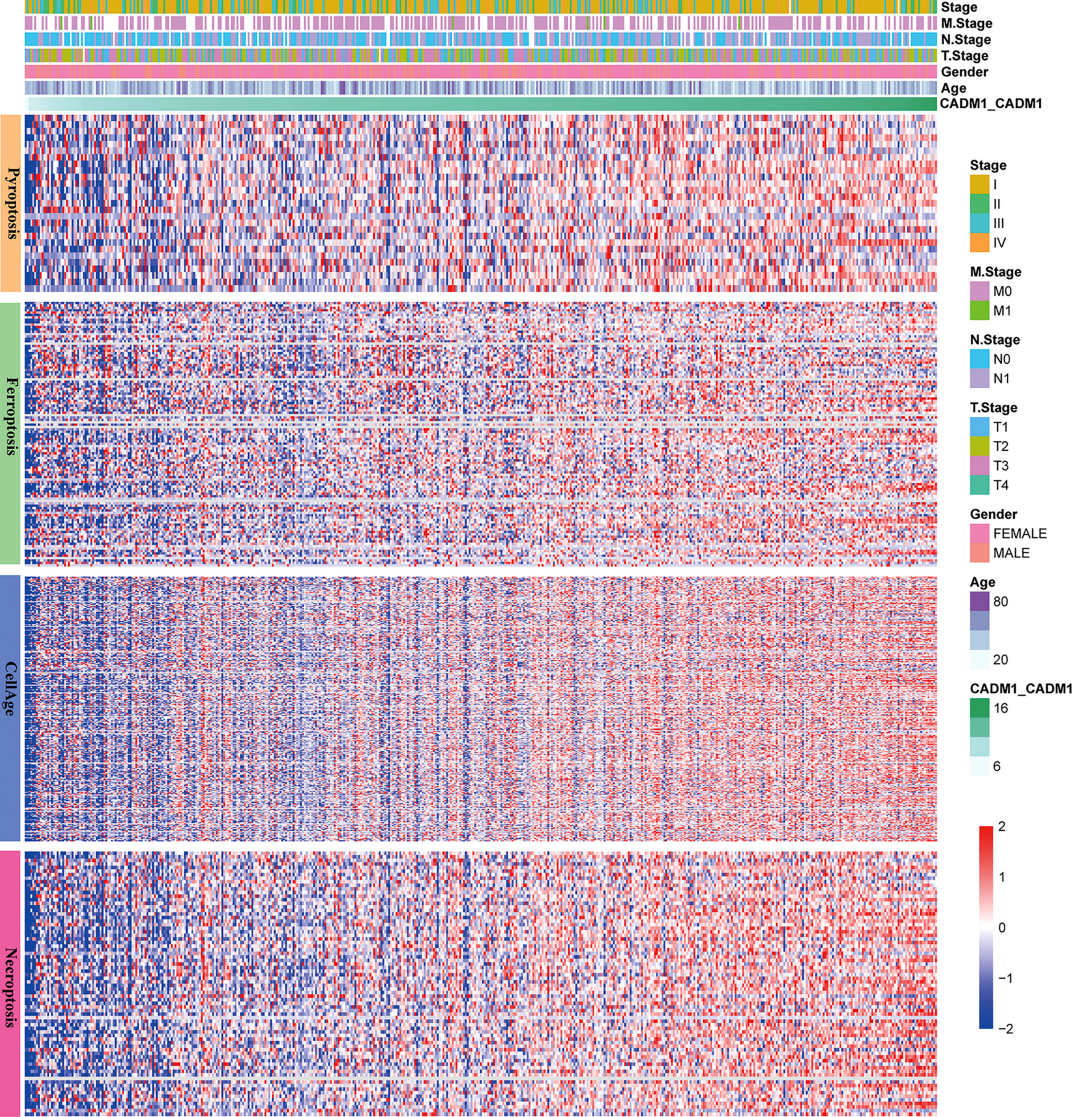
The relationship between CADM1-CADM1, gender, age, stage, T stage, M stage, N stage and genes associated with apoptosis, ferroptosis, pyroptosis and cellular senescence.

Apoptosis, ferroptosis, pyroptosis and cellular senescence adjust the development of tumor. The reduction of apoptosis can lead to malignant transformation of the affected cells, tumor metastasis and resistance to anticancer drugs (25). Ferroptosis, a newly discovered cell death form, was confirmed to play a pivotal role in killing tumor cells and suppressing tumor growth (26). Pyroptosis promotes inflammatory cell death of cancer and inhibit proliferation and migration of cancer cells (27). Cellular senescence can inhibit tumor proliferation but active tumorigenesis by extrinsically promoting evasion of the immune system (28). Thus, it is necessary to analysis the connection between CADM1-CADM1 and cell death. Through analyzing the connection between CADM1-CADM1 and genes associated with apoptosis, ferroptosis, pyroptosis and cellular senescence (**Figure 8**), we found the expression of these genes significantly increased with the up-regulation of CADM1-CADM1, which meant CADM1-CADM1 was involved in these biological processes.

### 3.8 Analysis of drug sensitivity targeted on CADM1-CADM1

**Figure 9:**
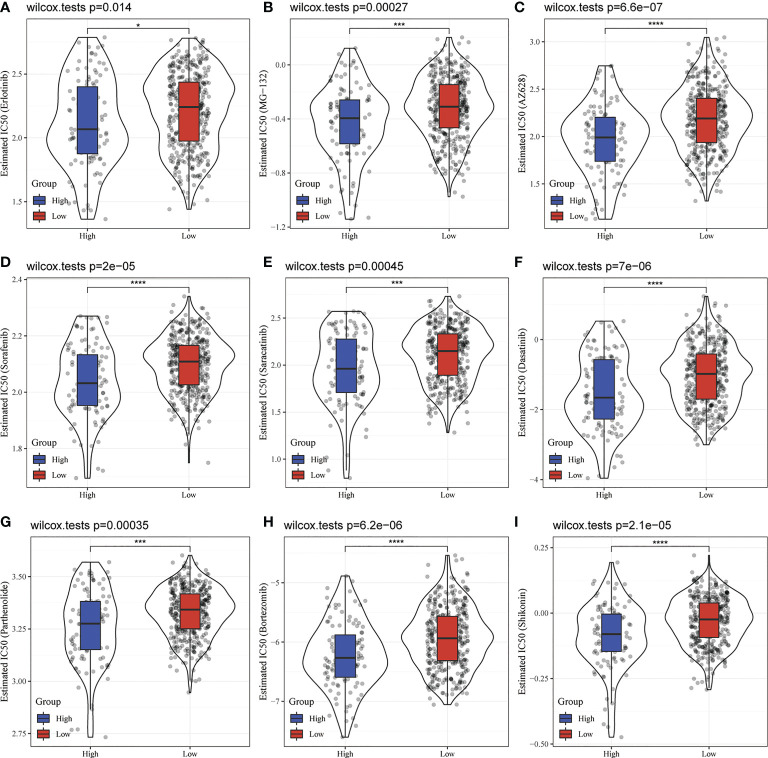
Comparison of targeted drug sensitivity in high CADM1-CADM1 group and low CADM1-CADM1 group. (****P < 0.0001, ***P < 0.001, *P < 0.05). **(A)** Comparison of the sensitivity of Erlotinib. **(B)** Comparison of the sensitivity of MG-132. **(C)** Comparison of the sensitivity of AZ628. **(D)** Comparison of the sensitivity of Sorafenib. **(E)** Comparison of the sensitivity of Saracatinib. **(F)** Comparison of the sensitivity of Dasatinib. **(G)** Comparison of the sensitivity of Parthenolide. **(H)** Comparison of the sensitivity of Bortezomib. **(I)** Comparison of the sensitivity of Shikonin.

No matter molecular mechanisms regulated or cell death involved by CADM1-CADM1, they all play an important role in tumor resistance, thus, we further studied the influence of CADM1-CADM1 on targeted drugs using pRRophetic package in R. Surprisingly, the high expression of CADM1-CADM1 strongly increased the sensitivity of many targeted drugs such as Erlotinib, MG-132, AZ628 (**Figure 9**), which meant therapeutic effects of targeted drugs could be improved by upregulating the expression of CADM1-CADM1.

### 3.9 The expression of CADM1-CADM1 in pan-cancer

**Figure 10:**
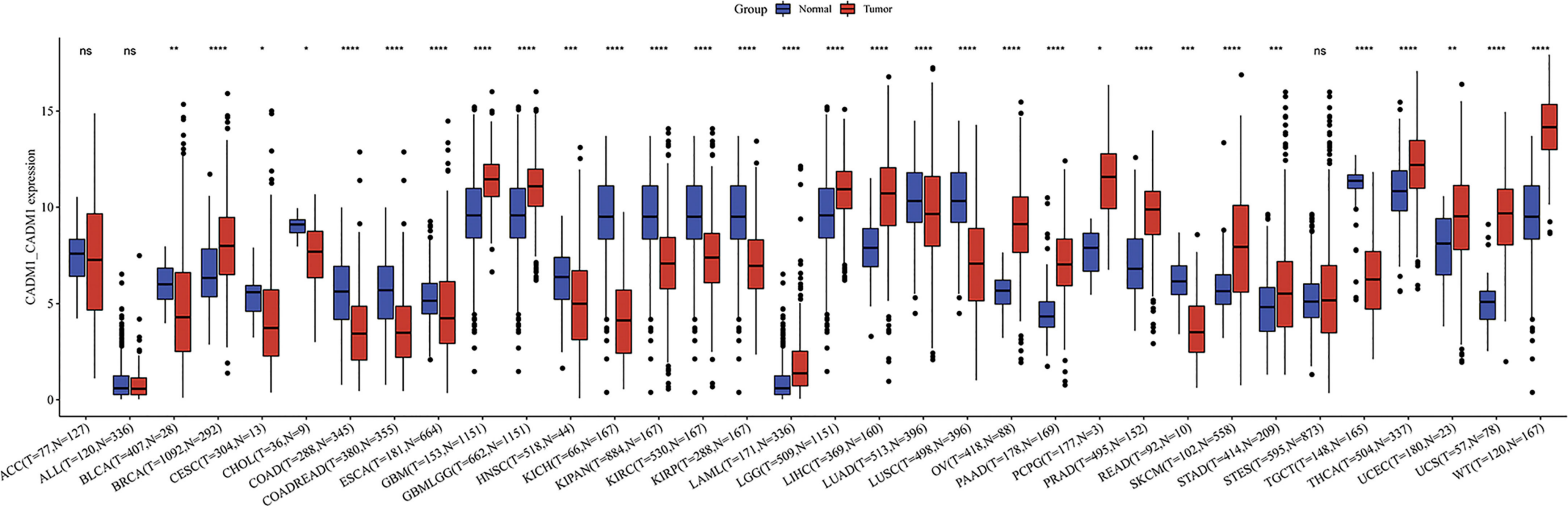
The differential expression of CADM1-CADM1 between tumor tissues and normal tissues in pan-cancer (****P < 0.0001, ***P < 0.001, **P < 0.01, *P < 0.05).

We compared the expression of CADM1-CADM1 in cancers and normal tissues, and the result showed that CADM1-CADM1 differently expressed in many cancers including up-regulation and down-regulation (**Figure 10**). The downregulated CADM1-CADM1 expression was observed consistently in tumor tissues versus normal tissues in the *BLCA, CESC, CHOL, COAD, COADREAD, ESCA, HNSC, KICH, KIPAN, KIRC, KIRP, LAML, LUAD, LUSC, OV, READ* and *TGCT* datasets. However, CADM1-CADM1 was expressed higher in tumor tissues than normal tissues in the *BRCA, GBM, GBMLGG, LGG, LIHC, PAAD, PCPG, PRAD, SKCM, STAD, STES, THCA, UCEC, UCS and WT* datasets.

### 3.10 qRT-PCR and transwell assay

**Figure 11:**
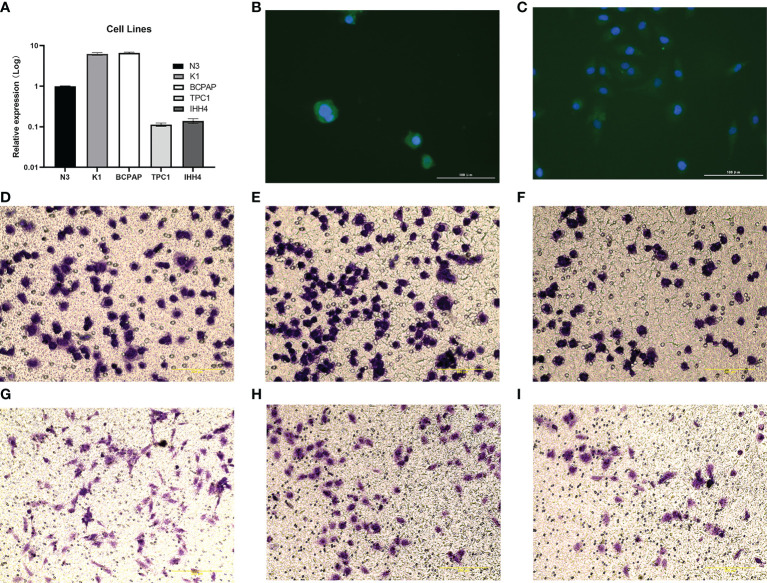
The expression of CADM1-CADM1 in PTC cell lines. **(A)** The different expression of CADM1 in N3, K1, BAPAP, TPC1 and IHH4 cell lines. **(B)** Results of immunofluorescence staining of IHH4 cells, which were mainly located in cytoplasm and a little in nucleus. **(C)** Results of immunofluorescence staining of TPC1 cells, which were also mainly located in cytoplasm and a little in nucleus. **(D–I)** Overexpression of CADM1 in IHH4 cell lines by pEGFP-C1-CADM1 transfection inhibits cell migration. D, Blank control (IHH4) (×200). E, pEGFP-C1 (NC) (×200). F, pEGFP-C1-CADM1 (CADM1) (×200). **(G–I)** Overexpression of CADM1 in TPC1 cell lines by pEGFP-C1-CADM1 transfection inhibits cell migration. G, Blank control (TPC1) (×200).H, pEGFP-C1 (NC) (×200). I, pEGFP-C1-CADM1 (CADM1) (×200).

As shown in **Figure 11A**, CADM1 was differently expressed in K1, BCPAP, TPC1 and IHH4 cancer cell lines, among which CADM1 was lowly expressed in IHH4 and TPC1. As we can see, the CADM1 protein was mainly localized in the cytoplasm of cells, and also partially expressed in the nucleus (**Figures 11B, C**). As shown in **Figures 11D–F**, CADM1 overexpression blocked IHH4 cell migration, the mean ± SD number of migrating cells was 110.0 ± 5.5 in the IHH4 cell line group, 105.5 ± 10.2 in the pEGFP-C1 (used as a negative control) group and 75.5 ± 7.8 in the pEGFP-C1-CADM1 group. In **Figures 11G–I**, the mean ± SD number of migrating cells was 92.5± 10.1 in the TPC1 cell line group, 98.1 ± 8.5 in the pEGFP-C1 group and 47.7 ± 5.6 in the pEGFP-C1-CADM1 group, which was in consist with the analysis above and meant CADM1 could be a therapeutic target for advanced PTC.

## 4 Discussion

Advanced PTC has a bad prognosis, but the assessment and treatment of advanced PTC still not clear. With more studies focused on TME, we ensure the interactions between TME and tumor based on L/R pairs are strongly associated with the progression of tumor. More and more therapies targeting L/R pairs have been applied on advanced tumors. However, quite a few studies focused on L/R pairs was performed in PTC. Thus, it is interesting and necessary to explore L/R pairs to provide individualized and precise treatment for PTC, particularly advanced PTC. In our study, the L/R pair: CADM1-CADM1 was screened through scRNA-seq, bulk RNA-seq and differential analysis. We found it might be used to be a biomarker to assess the prognosis of PTC and a therapeutic target for advanced PTC.

CADM1-CADM1 mainly distributed in malignant-malignant, malignant-thyrocytes, thyrocytes-malignant. The GO enrichment results showed CC was mainly enriched in ‘collagen-containing extracellular matrix’, ‘receptor complex’, and ‘extracellular matrix’. Previous studies have shown CADM1, a member of the IgSF and expressed in the cytoplasm and cell membrane (21, 29). CADM1 is a single-pass, type 1 membrane protein composed of three extracellular immunoglobulin-like domains, a single transmembrane region, and a short carboxyl terminal intracellular tail (30), which is consistent with the result of GO enrichment analysis. Besides, our result identified that CADM1-CADM1 could assess the prognosis of PTC, the high expression of which would bring a long survival time. In the meanwhile, our results showed that the high expression of CADM1-CADM1 could inhibit the invasion of primary tumor but also showed that it would increase the metastasis of LNM. Given that LNM is a complex process that the mechanism is unclear, we thought LNM might be influenced by other factors. Interestingly, the BP terms in GO enrichment analysis enriched in ‘blood vessel morphogenesis’, which have been reported could be inhibited by up-regulating the expression of CADM1 (31, 32). Some studies which focused on CADM1 expressed in other epithelial cell tumors also support our views: G. Zhang et al. reported that down-regulating CADM1 would lead to the progression of breast cancer (33); X. Si et al. showed that overexpression of CADM1 could inhibit ovarian cancer cell proliferation and migration (34); Z. Yang et al. proved that the down-regulation of CADM1 enhanced migration and invasion in GC cells (35). However, for all we know, there is no previous study focused on the relationship between CADM1-CADM1 and PTC. Our study also showed the expression of CADM1-CADM1 could be modulated by the degree of DNA hypermethylation in PTC, which increased the possibility to become a practical clinical biomarker for PTC. Some studies show that CADM1 could be used as a biomarker for cervical lesions and malignant melanoma (36, 37).

Furthermore, CADM1-CADM1 regulated many pathways associated with the tumor cell proliferation, metastasis and death. The GO analysis results revealed that CADM1-CADM1 was connected with ‘Ras guanyl-nucleotide exchange factor activity’ in MF. Ras guanyl-nucleotide exchange factor activates the conversion of inactive RAS-GDP to active RAS-GTP, which leads to the activation of MAPK signaling pathway and PI3K-Akt signaling pathway (38). The PI3K-Akt signaling pathway can suppresses the Bax activation-induced apoptosis by promoting HK-2 translocation to mitochondria (39). The activation of MAPK signaling pathway would lead to tumor proliferation, metastasis. Ras guanyl-nucleotide exchange factor can also activate Rap 1, which can increase cell viability and cell–matrix adhesion (40) and inhibit apoptosis by suppressing tumor necrosis factor-α induced-reactive oxygen species (ROS) production (39). Thus, we thought that the high expression of CADM1-CADM1 would downregulate the activation of Ras guanyl-nucleotide exchange factor, so as to suppress the progression of PTC. Meanwhile, H. et al. reported the downregulation of CADM1 was able to activate the P44/42 MAPK signaling pathway associated with cell proliferation, migration and invasion (41), and CADM1 acts as an inhibitor of the RAS-RAF-MEK1/2-ERK1/2 pathway to inhibit the EMT in melanoma (42),which are consistent with our thought. However, some studies expound that the activity of MAPK signaling pathway can active ferroptosis (43, 44), which can kill tumor cells and suppress tumor growth. This is inconsistent with our thought. Besides MAPK signaling pathway, Murakami et al. demonstrated that trans-homophilic interactions, mediated by CADM1, activated the PI3K pathway to reorganize the actin cytoskeleton and form the epithelial cell structure, which inhibit tumor progression (23, 34), Y. Wang et al. stated overexpression of TSLC1 downregulated the transcriptional activity of TCF4/β-catenin and inhibited the mRNA or protein expression of Wnt target genes cyclinD1 and c-myc, which could suppress tumor cell migration, invasion, apoptosis (45). Taken together, the pathways regulated by CADM1-CADM1 are complex and they are important for the tumor biological behaviors: proliferate, death, invasion and metastasize, which inhibit the progression of tumor totally.

In our study, we identified that the expression of CADM1-CADM1 in PTC can increase the sensitivity of many targeted drugs. Related researches showed that failure to up-regulate CADM1 in response to chemotherapeutic drugs may contribute to therapy resistance in AML (46) and CADM1 sensitizes tongue cancer cells to chemotherapy (47). Given conclusion above and our results, up-regulating the expression of CADM1-CADM1 in PTC would be beneficial to targeted therapies in advanced PTC. The expression of CADM1-CADM1 was down-regulated by DNA hypermethylation. Meanwhile, the KEGG enrichment analysis only enriched in one pathway: microRNA in cancer. The microRNA in cancer can strongly modulate cell circle, programmed cell death, invasion and metastasis (48), which is due to deregulated in tumor. MicroRNA itself chromosomal abnormality can lead to deregulation. Apart from this, hypermethylation of CpG islands in promoter regions results in heritable transcriptional silencing of tumor-suppressor genes in many cancers through repressing miRNAs (48). Therefore, inhibiting DNA methylation and modulating associated miRNAs are useful for up-regulating the expression of CADM1-CADM1 in PTC. At present, large number of DNMT inhibitor candidates which can inhibit DNA methylation are in the developmental pipeline and some are currently in clinical trials (49). Although there is no study on increasing the expression of CADM1 through regulating associated miRNAs in PTC up to now, there are some studies on other cancers: inhibiting microRNA-1246 can increase the expression of CADM1 in hepatocellular carcinoma (50); upregulating miR-486 can downregulate CADM1 expression in Ovarian Cancer (51); miR-155-3p could downregulate expression of CADM1 in breast cancer (33). Thus, more researches are needed to explore related miRNAs in PTC. In addition, the way that CADM1 performs functions is combined to the CADM1 receptor, thus, mimicking CADM1 acting the CADM1 receptor may be useful to increase the sensitivity of many targeted drugs. What’s more, the prognosis and survival rate of cancer patients remain unsatisfactory due to drug resistance, side effects, and other problems (52), the differential expression of CADM1-CADM1 in pan-cancer may provide new viewpoints for personalized treatment strategies of cancer.

Although we identified the L/R pair: CADM1-CADM1 could be a biomarker for the prognosis and treatment of PTC, there are several limitations existed in the current study. In order to further ensure the prognostic value of CADM1-CADM1, more prospective studies are required. The pathways regulated by CADM1-CADM1 should be proven *in vitro* experiments. What’s more important, targeted therapies on CADM1-CADM1 should be further explored.

## 5 Conclusion

In summary, we first proposed the L/R pair: CADM1-CADM1 played an essential role in the progression of PTC, which can be used to assess the prognosis of PTC. It is worth noticing that the high expression of CADM1-CADM1 can increase the sensitivity of many targeted drugs, which can be helpful to alleviate drug resistance and provide a novel target for advanced PTC.

## Data availability statement

The datasets presented in this study can be found in online repositories. The names of the repository/repositories and accession number(s) can be found in the article/**Supplementary Material**.

## Author contributions

Conception and design: HH. Acquisition of data: HH and SC. Analysis and interpretation of data: HH, SC and WL. Writing, review, and revision of the manuscript: HH, SC, YW, and QJ Study supervision: NQ. All authors read and approved the final manuscript.

## Funding

This study was supported by the Natural Science Foundation of China (No. 81903964 to HH); Shanghai Science and Technology Commission Research Projects (No. 201840268 to WL)

## Conflict of interest

The authors declare that the research was conducted in the absence of any commercial or financial relationships that could be construed as a potential conflict of interest.

## Publisher’s note

All claims expressed in this article are solely those of the authors and do not necessarily represent those of their affiliated organizations, or those of the publisher, the editors and the reviewers. Any product that may be evaluated in this article, or claim that may be made by its manufacturer, is not guaranteed or endorsed by the publisher.

## Supplementary material

Supplementary Figure 1 scRNA−seq data processing. **(A)** The relationship between mitochondrial genes and UMI/mRNA quantity, the relationship between UMI and mRNA quantity; **(B)** The relationship between the mRNA/UMI/mitochondrial content/rRNA content of each sample before filtering; **(C)** The relationship between mRNA/UMI/mitochondrial content/rRNA content of each sample after filtering; **(D)** The sample distribution map of PCA dimensionality reduction and the anchor point map of PCA.

Supplementary Figure 2 t-SNE projection showing the expression of marker genes in 9 cell clusters.

Supplementary Figure 3 Network plot diagram shows ligand-receptor pair interactions.

Supplementary Figure 4 DFI KM curve of CADM1-CADM1.

Supplementary Figure 5 PFI KM curve of CADM1-CADM1.

Supplementary Table 1 Differential analysis of 16 L/R pairs in detail.

Supplementary Table 2 Univariate Cox regression analysis of 16 L/R pairs.

Supplementary Table 3 Differential genes associated with CADM1-CADM in TCGA.

Supplementary Table 4 279 positively correlated genes and 2 negatively correlated genes.

Supplementary Table 5 The results of KEGG and GO enrichment analysis.

Supplementary Table 6 The distribution of CADM1-CADM1.

## References

[1] Seib CD Sosa JA . Evolving understanding of the epidemiology of thyroid cancer. Endocrinol Metab Clin North Am (2019) 48:23–35. doi: 10.1016/j.ecl.2018.10.002 30717905

[2] Sung H Ferlay J Siegel RL Laversanne M Soerjomataram I Jemal A . Global cancer statistics 2020: GLOBOCAN estimates of incidence and mortality worldwide for 36 cancers in 185 countries. CA Cancer J Clin (2021) 71:209–49. doi: 10.3322/caac.21660 33538338

[3] Dettmer MS Schmitt A Steinert H Capper D Moch H Komminoth P . Tall cell papillary thyroid carcinoma: new diagnostic criteria and mutations in BRAF and TERT. Endocr Relat Cancer (2015) 22:419–29. doi: 10.1530/ERC-15-0057 25870252

[4] Eustatia-Rutten CF Corssmit EP Biermasz NR Pereira AM Romijn JA Smit JW . Survival and death causes in differentiated thyroid carcinoma. J Clin Endocrinol Metab (2006) 91:313–9. doi: 10.1210/jc.2005-1322 16263822

[5] Fugazzola L Elisei R Fuhrer D Jarzab B Leboulleux S Newbold K . 2019 European thyroid association guidelines for the treatment and follow-up of advanced radioiodine-refractory thyroid cancer. Eur Thyroid J (2019) 8:227–45. doi: 10.1159/000502229 PMC6873012 31768334

[6] Karapanou O Simeakis G Vlassopoulou B Alevizaki M Saltiki K . Advanced RAI-refractory thyroid cancer: an update on treatment perspectives. Endocr Relat Cancer (2022) 29(5):R57–R66. doi: 10.1530/erc-22-0006 35266878

[7] Enomoto K Inohara H . Surgical strategy of locally advanced differentiated thyroid cancer. Auris Nasus Larynx (2022) 22:00063–3. doi: 10.1016/j.anl.2022.03.005 35314084

[8] Spill F Reynolds DS Kamm RD Zaman MH . Impact of the physical microenvironment on tumor progression and metastasis. Curr Opin Biotechnol (2016) 40:41–8. doi: 10.1016/j.copbio.2016.02.007 PMC4975620 26938687

[9] Del Prete A Schioppa T Tiberio L Stabile H Sozzani S . Leukocyte trafficking in tumor microenvironment. Curr Opin Pharmacol (2017) 35:40–7. doi: 10.1016/j.coph.2017.05.004 28577499

[10] Arneth B . Tumor microenvironment. Medicina (2019) 56(1):15–36. doi: 10.3390/medicina56010015 PMC7023392 31906017

[11] Camp JG Sekine K Gerber T Loeffler-Wirth H Binder H Gac M . Multilineage communication regulates human liver bud development from pluripotency. Nature (2017) 546:533–8. doi: 10.1038/nature22796 28614297

[12] Ramilowski JA Goldberg T Harshbarger J Kloppmann E Lizio M Satagopam VP . A draft network of ligand-receptor-mediated multicellular signalling in human. Nat Commun (2015) 6:7866. doi: 10.1038/ncomms8866 26198319 PMC4525178

[13] Hanahan D Weinberg RA . Hallmarks of cancer: the next generation. Cell (2011) 144:646–74. doi: 10.1016/j.cell.2011.02.013 21376230

[14] Lin H Xia L Lian J Chen Y Zhang Y Zhuang Z . Delineation of colorectal cancer ligand-receptor interactions and their roles in the tumor microenvironment and prognosis. J Transl Med (2021) 19:497. doi: 10.1186/s12967-021-03162-0 34876143 PMC8650275

[15] Buenrostro JD Wu B Litzenburger UM Ruff D Gonzales ML Snyder MP . Single-cell chromatin accessibility reveals principles of regulatory variation. Nature (2015) 523:486–90. doi: 10.1038/nature14590 PMC4685948 26083756

[16] Melaiu O Lucarini V Cifaldi L Fruci D . Influence of the tumor microenvironment on NK cell function in solid tumors. Front Immunol (2019) 10:3038. doi: 10.3389/fimmu.2019.03038 32038612 PMC6985149

[17] Tao L Qiu J Slavin S Ou Z Liu Z Ge J . Recruited T cells promote the bladder cancer metastasis *via* up-regulation of the estrogen receptor beta/IL-1/c-MET signals. Cancer Lett (2018) 430:215–23. doi: 10.1016/j.canlet.2018.03.045 29684419

[18] Chen X Cubillos-Ruiz JR . Endoplasmic reticulum stress signals in the tumour and its microenvironment. Nat Rev Cancer (2021) 21:71–88. doi: 10.1038/s41568-020-00312-2 33214692 PMC7927882

[19] Wu Y Li N Ye C Jiang X Luo H Zhang B . Focal adhesion kinase inhibitors, a heavy punch to cancer. Discovery Oncol (2021) 12:52. doi: 10.1007/s12672-021-00449-y PMC8777493 35201485

[20] Liu J Lichtenberg T Hoadley KA Poisson LM Lazar AJ Cherniack AD . An integrated TCGA pan-cancer clinical data resource to drive high-quality survival outcome analytics. Cell (2018) 173:400–416 e411. doi: 10.1016/j.cell.2018.02.052 29625055 PMC6066282

[21] Takai Y Ikeda W Ogita H Rikitake Y . The immunoglobulin-like cell adhesion molecule nectin and its associated protein afadin. Annu Rev Cell Dev Biol (2008) 24:309–42. doi: 10.1146/annurev.cellbio.24.110707.175339 18593353

[22] Ali Syeda Z Langden SSS Munkhzul C Lee M Song SJ . Regulatory mechanism of MicroRNA expression in cancer. Int J Mol Sci (2020) 21(5):1723–41. doi: 10.3390/ijms21051723. PMC7084905 32138313

[23] Murakami S Sakurai-Yageta M Maruyama T Murakami Y . Trans-homophilic interaction of CADM1 activates PI3K by forming a complex with MAGuK-family proteins MPP3 and dlg. PloS One (2014) 9:e82894. doi: 10.1371/journal.pone.0082894 24503895 PMC3913574

[24] Zhang R Tan Z Liang P . Identification of a novel ligand-receptor pair constitutively activated by ras oncogenes. J Biol Chem (2000) 275:24436–43. doi: 10.1074/jbc.M001958200 10825166

[25] Wong RS . Apoptosis in cancer: from pathogenesis to treatment. J Exp Clin Cancer Res (2011) 30:87. doi: 10.1186/1756-9966-30-87 21943236 PMC3197541

[26] Yu H Guo P Xie X Wang Y Chen G . Ferroptosis, a new form of cell death, and its relationships with tumourous diseases. J Cell Mol Med (2017) 21:648–57. doi: 10.1111/jcmm.13008 PMC5345622 27860262

[27] Fang Y Tian S Pan Y Li W Wang Q Tang Y . Pyroptosis: A new frontier in cancer. Biomed Pharmacother (2020) 121:1–7. doi: 10.1016/j.biopha.2019.109595 31710896

[28] Prieto LI Baker DJ . Cellular senescence and the immune system in cancer. Gerontology (2019) 65:505–12. doi: 10.1159/000500683 PMC6703936 31212284

[29] Nakahata S Morishita K . CADM1/TSLC1 is a novel cell surface marker for adult T-cell leukemia/lymphoma. J Clin Exp Hematop (2012) 52:17–22. doi: 10.3960/jslrt.52.17 22706526

[30] De Maria A Shi Y Luo X van der Weyden L Bassnett S . Cadm1 expression and function in the mouse lens. Invest Opthalmol Visual Sci (2011) 52(5):2293–99. doi: 10.1167/iovs.10-6677 PMC3080734 21217103

[31] Zhang F Liu J Xie BB . Downregulation of microRNA-205 inhibits cell invasion and angiogenesis of cervical cancer through TSLC1-mediated akt signaling pathway. J Cell Physiol (2019) 234:18626–38. doi: 10.1002/jcp.28501 31049956

[32] Kocher AA Schuster MD Szabolcs MJ Takuma S Burkhoff D Wang J . Neovascularization of ischemic myocardium by human bone-marrow-derived angioblasts prevents cardiomyocyte apoptosis, reduces remodeling and improves cardiac function. Nat Med (2001) 7:430–6. doi: 10.1038/86498 11283669

[33] Zhang G Zhong L Luo H Wang S . MicroRNA-155-3p promotes breast cancer progression through down-regulating CADM1. Onco Targets Ther (2019) 12:7993–8002. doi: 10.2147/OTT.S206180 31579252 PMC6773971

[34] Si X Xu F Xu F Wei M Ge Y Chenge S . CADM1 inhibits ovarian cancer cell proliferation and migration by potentially regulating the PI3K/Akt/mTOR pathway. BioMed Pharmacother (2020) 123:109717. doi: 10.1016/j.biopha.2019.109717 31865146

[35] Yang Z Wang R Zhang T Dong X . MicroRNA-126 regulates migration and invasion of gastric cancer by targeting CADM1. Int J Clin Exp Pathol (2015) 8:8869–80. PMC4583860 26464628

[36] Del Pino M Sierra A Marimon L Martí Delgado C Rodriguez-Trujillo A Barnadas E . CADM1, MAL, and miR124 promoter methylation as biomarkers of transforming cervical intrapithelial lesions. Int J Mol Sci (2019) 20(9):2262–76. doi: 10.3390/ijms20092262 PMC6539131 31067838

[37] El Aliani A El-Abid H El Mallali Y Attaleb M Ennaji MM and El Mzibri M . Association between gene promoter methylation and cervical cancer development: Global distribution and a meta-analysis. Cancer Epidemiol Biomarkers Prev (2021) 30:450–9. doi: 10.1158/1055-9965.EPI-20-0833 33441308

[38] Roskoski R Jr . Blockade of mutant RAS oncogenic signaling with a special emphasis on KRAS. Pharmacol Res (2021) 172:105806. doi: 10.1016/j.phrs.2021.105806 34450320

[39] Takino JI Sato T Nagamine K Hori T . The inhibition of bax activation-induced apoptosis by RasGRP2 *via* r-Ras-PI3K-Akt signaling pathway in the endothelial cells. Sci Rep (2019) 9:16717. doi: 10.1038/s41598-019-53419-4 31723205 PMC6854084

[40] Takino J Nagamine K Hori T . Ras guanyl nucleotide releasing protein 2 affects cell viability and cell-matrix adhesion in ECV304 endothelial cells. Cell Adh Migr (2013) 7:262–6. doi: 10.4161/cam.24082 PMC3711991 23563504

[41] Cai H Miao M Wang Z . miR-214-3p promotes the proliferation, migration and invasion of osteosarcoma cells by targeting CADM1. Oncol Lett (2018) 16:2620–8. doi: 10.3892/ol.2018.8927 PMC6036594 30013657

[42] Hartsough EJ Weiss MB Heilman SA Purwin TJ Kugel CH 3rd, Rosenbaum SR . CADM1 is a TWIST1-regulated suppressor of invasion and survival. Cell Death Dis (2019) 10:281. doi: 10.1038/s41419-019-1515-3 30911007 PMC6433918

[43] Wang X Zhang C Zou N Chen Q Wang C Zhou X . Lipocalin-2 silencing suppresses inflammation and oxidative stress of acute respiratory distress syndrome by ferroptosis *via* inhibition of MAPK/ERK pathway in neonatal mice. Bioengineered (2022) 13:508–20. doi: 10.1080/21655979.2021.2009970 PMC8805876 34969358

[44] Nguyen THP Mahalakshmi B Velmurugan BK . Functional role of ferroptosis on cancers, activation and deactivation by various therapeutic candidates-an update. Chem Biol Interact (2020) 317:108930. doi: 10.1016/j.cbi.2019.108930 31866335

[45] Wang Y Huang P Hu Y Guo K Jia X Huang B . An oncolytic adenovirus delivering TSLC1 inhibits wnt signaling pathway and tumor growth in SMMC-7721 xenograft mice model. Acta Biochim Biophys Sin (Shanghai) (2021) 53:766–74. doi: 10.1093/abbs/gmab048 33928346

[46] Fisser MC Rommer A Steinleitner K Heller G Herbst F Wiese M . Induction of the proapoptotic tumor suppressor gene cell adhesion molecule 1 by chemotherapeutic agents is repressed in therapy resistant acute myeloid leukemia. Mol Carcinog (2015) 54:1815–9. doi: 10.1002/mc.22252 25491945

[47] Zheng G Li N Jia X Peng C Luo L Deng Y . MYCN-mediated miR-21 overexpression enhances chemo-resistance *via* targeting CADM1 in tongue cancer. J Mol Med (Berl) (2016) 94:1129–41. doi: 10.1007/s00109-016-1417-0 27055844

[48] Lee YS Dutta A . MicroRNAs in cancer. Annu Rev Pathol: Mech Dis (2009) 4:199–227. doi: 10.1146/annurev.pathol.4.110807.092222 PMC2769253 18817506

[49] Uddin MG Fandy TE . DNA Methylation inhibitors: Retrospective and perspective view. Adv Cancer Res (2021) 152:205–23. doi: 10.1016/bs.acr.2021.03.007 PMC10275377 34353438

[50] Sun Z Meng C Wang S Zhou N Guan M Bai C . MicroRNA-1246 enhances migration and invasion through CADM1 in hepatocellular carcinoma. BMC Cancer (2014) 14:616. doi: 10.1186/1471-2407-14-616 25159494 PMC4150976

[51] Li C Wang Y Wang H Wang B Wang Y Li N . miR-486 promotes the invasion and cell cycle progression of ovarian cancer cells by targeting CADM1. Anal Cell Pathol (Amst) (2021) 2021:7407086. doi: 10.1155/2021/7407086 34395181 PMC8360751

[52] Siegel RL Miller KD Jemal A . Cancer statistics, 2019. CA Cancer J Clin (2019) 69:7–34. doi: 10.3322/caac.21551 30620402

