# Supplementary figures and images for: Analysis of the key ligand receptor CADM1_CADM1 in the regulation of thyroid cancer based on scRNA-seq and bulk RNA-seq data
Source: Front Endocrinol (Lausanne). 2022 Nov 29;13:969914. doi: 10.3389/fendo.2022.969914 (PMC9744787; doi:10.3389/fendo.2022.969914)

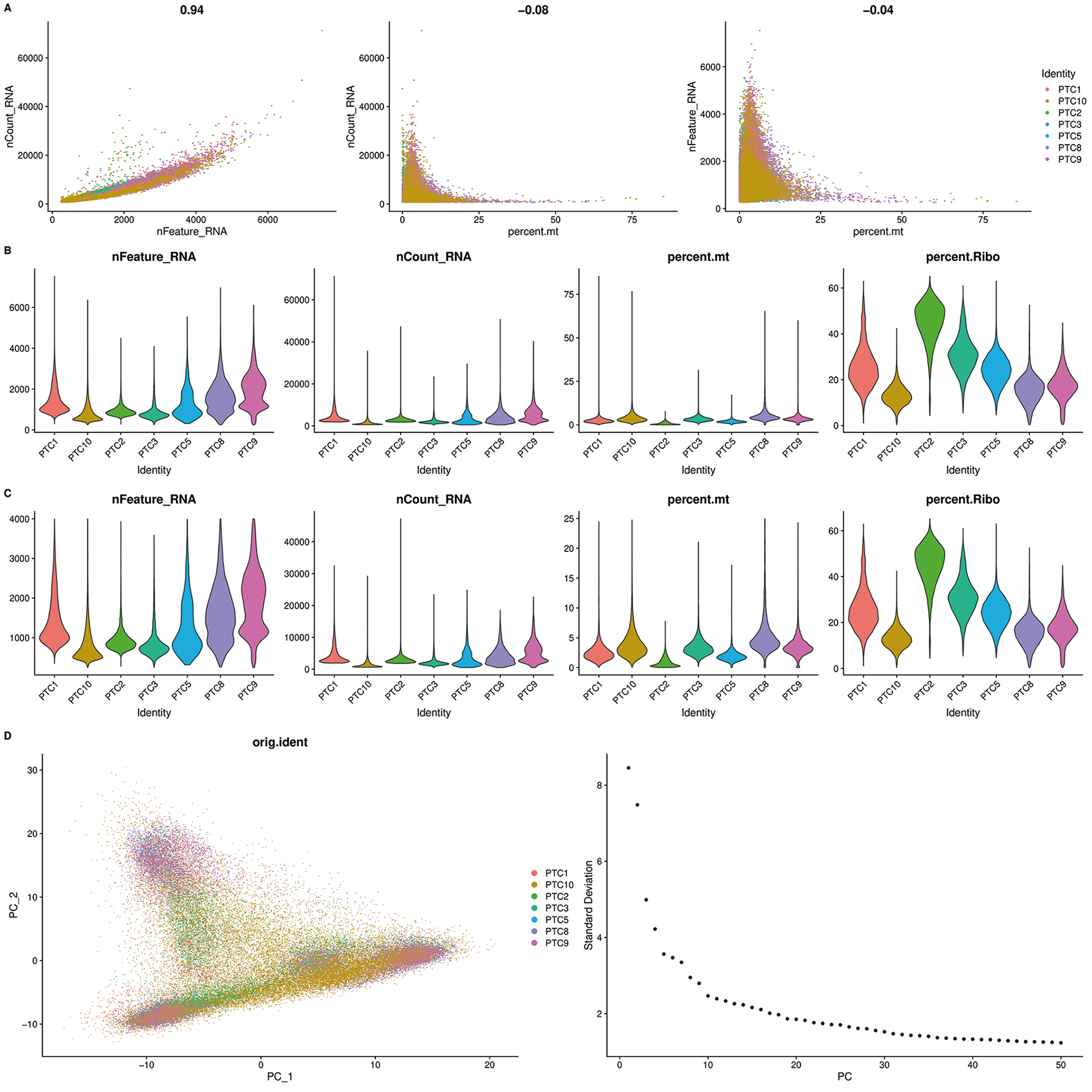

Supplement: Supplementary Figure 1 — scRNA−seq data processing. (A) The relationship between mitochondrial genes and UMI/mRNA quantity, the relationship between UMI and mRNA quantity; (B) The relationship between the mRNA/UMI/mitochondrial content/rRNA content of each sample before filtering; (C) The relationship between mRNA/UMI/mitochondrial content/rRNA content of each sample after filtering; (D) The sample distribution map of PCA dimensionality reduction and the anchor point map of PCA. [file DataSheet_1.zip › supplumentary meterial/Supplementary Fig 1.tif]

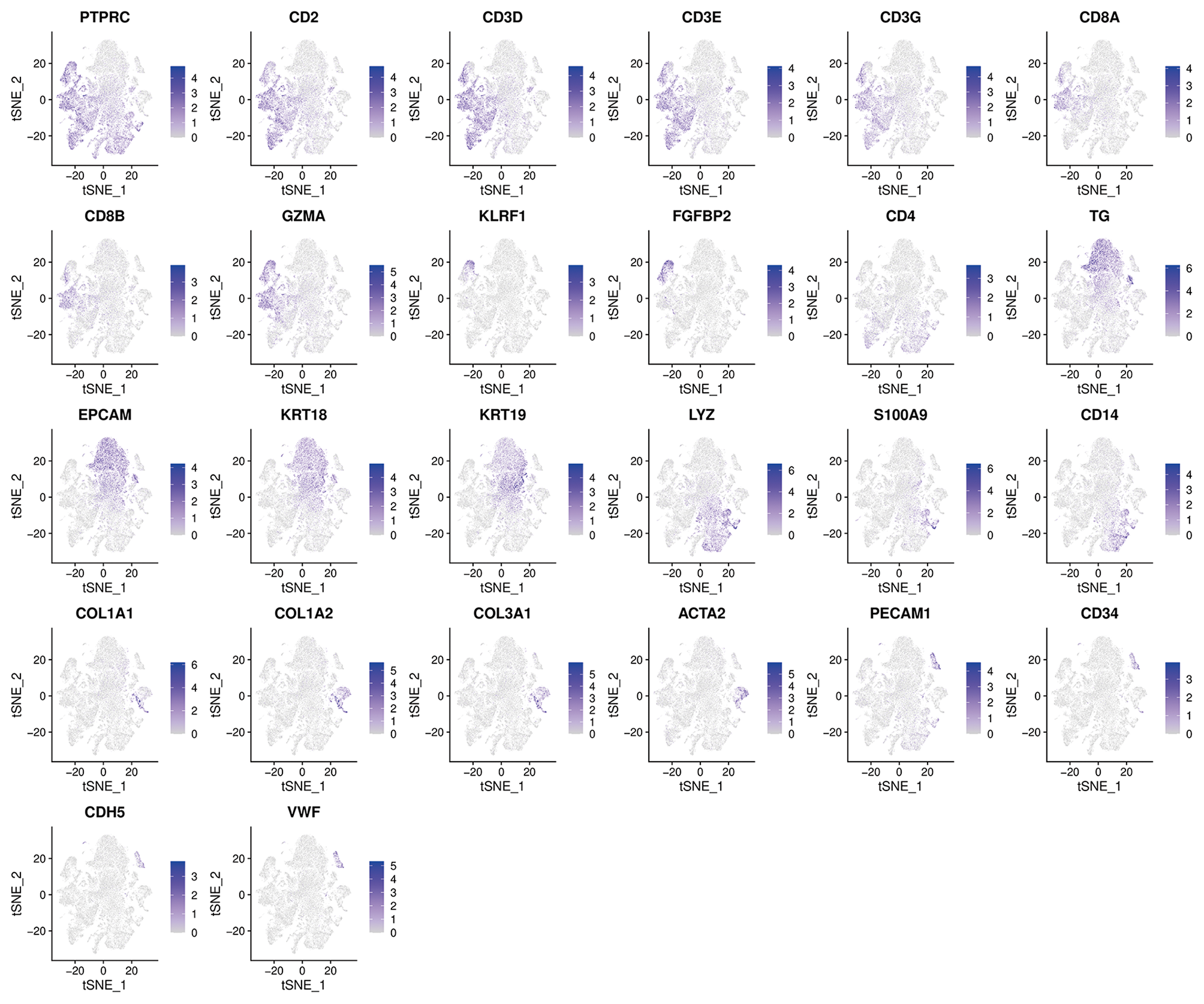

Supplement: Supplementary Figure 1 — scRNA−seq data processing. (A) The relationship between mitochondrial genes and UMI/mRNA quantity, the relationship between UMI and mRNA quantity; (B) The relationship between the mRNA/UMI/mitochondrial content/rRNA content of each sample before filtering; (C) The relationship between mRNA/UMI/mitochondrial content/rRNA content of each sample after filtering; (D) The sample distribution map of PCA dimensionality reduction and the anchor point map of PCA. [file DataSheet_1.zip › supplumentary meterial/Supplementary Fig 2.tif]

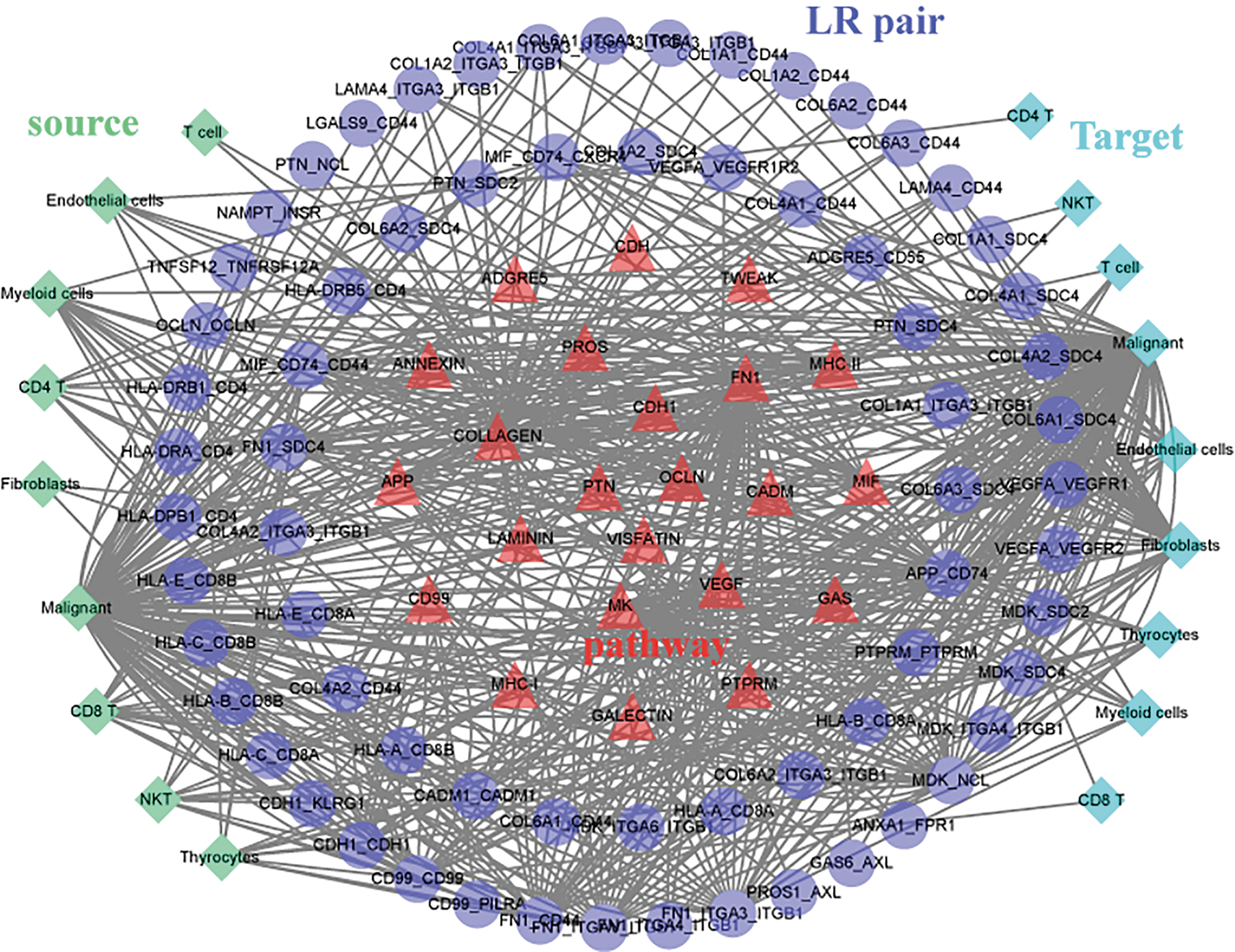

Supplement: Supplementary Figure 1 — scRNA−seq data processing. (A) The relationship between mitochondrial genes and UMI/mRNA quantity, the relationship between UMI and mRNA quantity; (B) The relationship between the mRNA/UMI/mitochondrial content/rRNA content of each sample before filtering; (C) The relationship between mRNA/UMI/mitochondrial content/rRNA content of each sample after filtering; (D) The sample distribution map of PCA dimensionality reduction and the anchor point map of PCA. [file DataSheet_1.zip › supplumentary meterial/Supplementary Fig 3.tif]

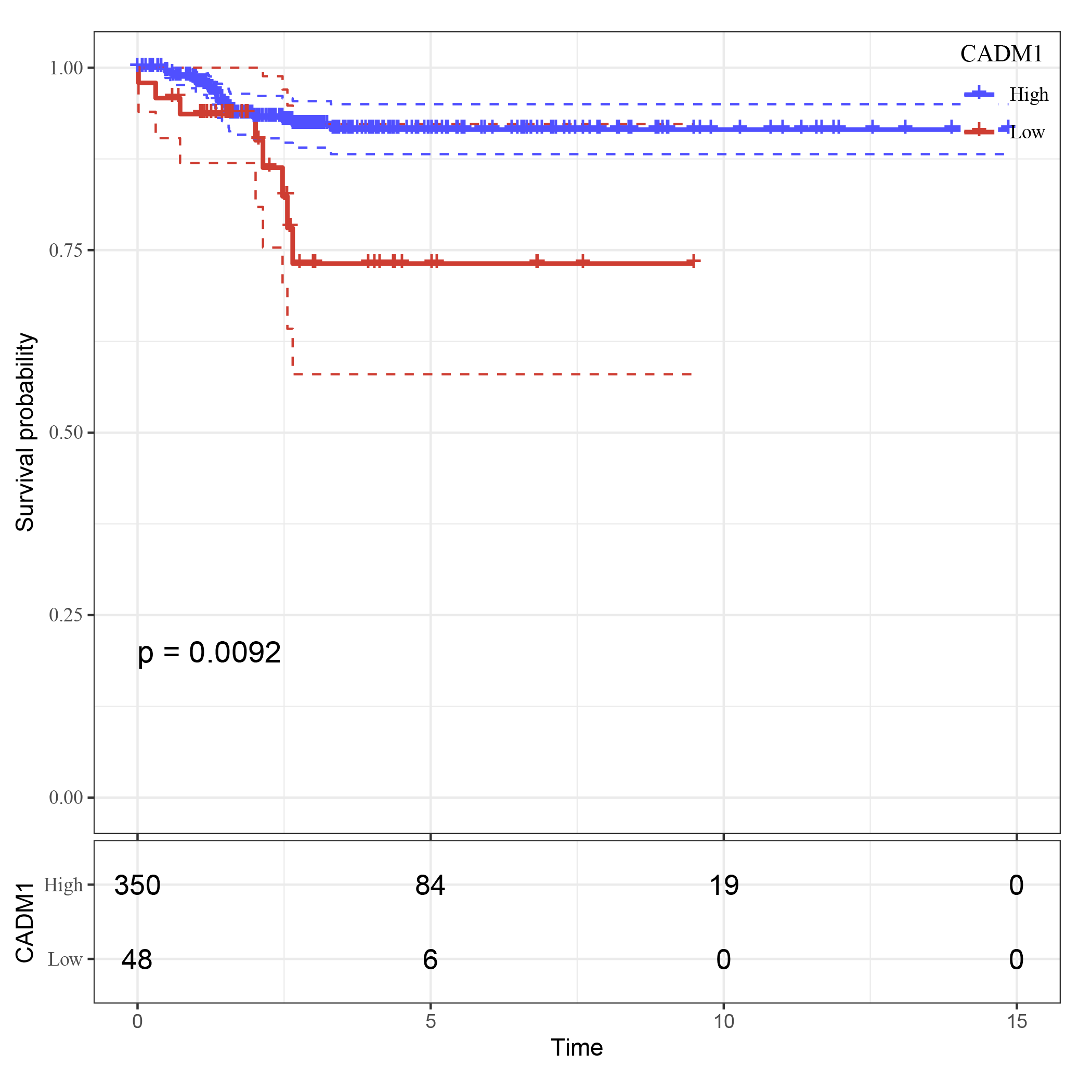

Supplement: Supplementary Figure 1 — scRNA−seq data processing. (A) The relationship between mitochondrial genes and UMI/mRNA quantity, the relationship between UMI and mRNA quantity; (B) The relationship between the mRNA/UMI/mitochondrial content/rRNA content of each sample before filtering; (C) The relationship between mRNA/UMI/mitochondrial content/rRNA content of each sample after filtering; (D) The sample distribution map of PCA dimensionality reduction and the anchor point map of PCA. [file DataSheet_1.zip › supplumentary meterial/Supplementary Fig 4.tif]

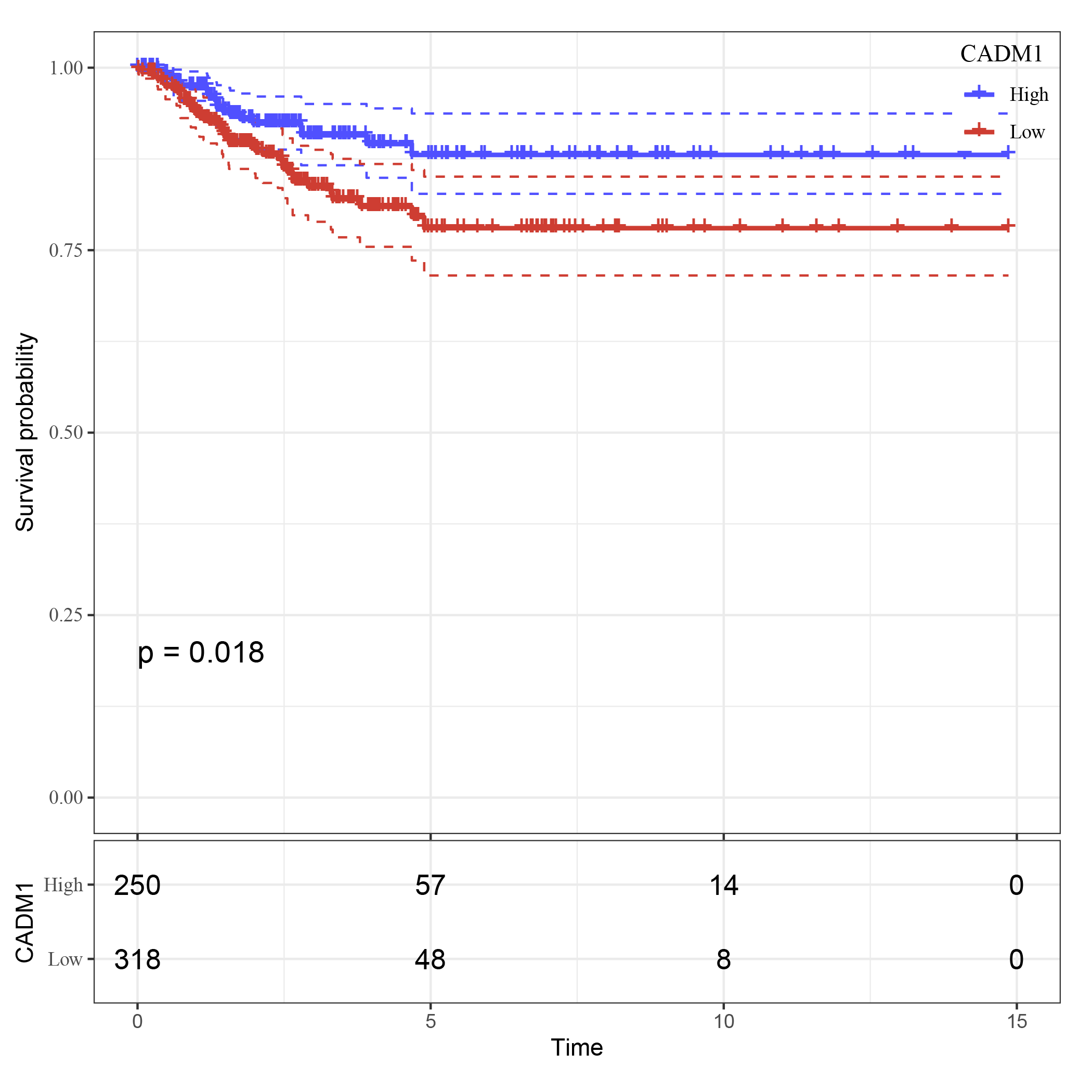

Supplement: Supplementary Figure 1 — scRNA−seq data processing. (A) The relationship between mitochondrial genes and UMI/mRNA quantity, the relationship between UMI and mRNA quantity; (B) The relationship between the mRNA/UMI/mitochondrial content/rRNA content of each sample before filtering; (C) The relationship between mRNA/UMI/mitochondrial content/rRNA content of each sample after filtering; (D) The sample distribution map of PCA dimensionality reduction and the anchor point map of PCA. [file DataSheet_1.zip › supplumentary meterial/Supplementary Fig 5.tif]
